# Supplementary material for: Quantitative sensing and signalling of single-stranded DNA during the DNA damage response
Source: Nat Commun. 2019 Feb 26;10:944. doi: 10.1038/s41467-019-08889-5 (PMC6391461; doi:10.1038/s41467-019-08889-5)
Supplement: Supplementary file 1 — Supplementary Information [file 41467_2019_8889_MOESM1_ESM.pdf]

Supplementary Information to

Quantitative sensing and signalling of single-stranded DNA during the DNA damage response

Bantele et al.

Contents:

Supplementary Tables 1-3
Supplementary Figures 1- 7
Supplementary Figure Legends

Supplementary Table 1. Yeast strains:

Strain	Relevant genotype	Fig. ref.	Source
W303a	MATa ade2-1 ura3-1 his3-11,15 trp1-1 leu2-3,112 can1-100		[1]
YSB5	MATa HML hmrΔ::pRS bar1Δ::trp1 pGal-HO::ade3	1A-E, F-G<br>2A,C-D<br>3A, I<br>S1A-E<br>S2C<br>S4A-B, D-G<br>S5A-G	this study
YSB147	MATa HML hmrΔ::pRS bar1Δ::trp1 pGal-HO::ade3 exo1Δ::natNT2 sgs1Δ::kanMX4	1A-B<br>S1B-D<br>S2C<br>S4F-G	this study
YSB87	MATa HML hmrΔ::pRS bar1Δ::trp1 pGal-HO::ade3 Ddc2-3FLAG::hphNT1	1A, D	this study
YSB380	MATa HML hmrΔ::pRS bar1Δ::trp1 pGal-HO::ade3 exo1Δ::natNT2 sgs1Δ::kanMX4 Ddc2-3FLAG::hphNT1	1A	this study
YSB633	MATa HML hmrΔ::pRS bar1Δ::trp1 HOcs-ChrIV::hphNT1 pGal-HO::ade3 exo1Δ::natNT2 sgs1Δ::kanMX4	1C<br>S2A-C	this study
YSB643	MATa HML hmrΔ::pRS bar1Δ::trp1 pGal-HO::ade3 HOcs-ChrIV::hphNT1	1C<br>S2A-C	this study
YSB336	MATa HML hmrΔ::pRS bar1Δ::trp1 pGal-HO::ade3 fun30Δ::kanMX4	1F-G	this study
YSB6	MATa HML hmrΔ::pRS bar1Δ::trp1 pGal-HO::ade3 rad9Δ::hphNT1	1F-G	this study
YSB1046	MATa HML hmrΔ::pRS bar1Δ::trp1 pGal-HO::ade3 fun30Δ::kanMX4 Ddc1-Fun30-3FLAG::hphNT1	1F-G	this study
YSB1064	MATa HML hmrΔ::pRS bar1Δ::trp1 pGal-	1F-G	this study

	HO::ade3 Yiplac128-Ddc1-Rad9-3FLAG::LEU2		
YSB165	MATa HML hmrΔ::pRS bar1Δ::trp1 pGal-HO::ade3 tel1Δ::hphNT1	2C<br>S4D-G<br>S5B-D	this study
YSB245	MATa HML hmrΔ::pRS bar1Δ::trp1 pGal-HO::ade3 sml1Δ::kanMX4 mec1Δ::hphNT1	2B<br>S4C-G	this study
YSB241	MATa HML hmrΔ::pRS bar1Δ::trp1 pGal-HO::ade3 sml1Δ::kanMX4	2B<br>S4C	this study
YSB1114	MATa HML hmrΔ::pRS bar1Δ::trp1 pGal-HO::ade3 sml1Δ::kanMX4 mec1Δ::hphNT1 tel1Δ::natNT2	S4D	this study
YSB374	MATa HML hmrΔ::pRS bar1Δ::trp1 pGal-HO::ade3 sml1Δ::kanMX4 mec1Δ::hphNT1 sgs1Δ::natNT2 exo1Δ::ura3	2B<br>S4C,E-G	this study
YSB371	MATa HML hmrΔ::pRS bar1Δ::trp1 pGal-HO::ade3 tel1Δ::hphNT1 sgs1Δ::natNT2 exo1Δ::ura3	2C<br>S4E-G	this study
YSB1098	MATa HML hmrΔ::pRS bar1Δ::trp1 pGal-HO::ade3 hta1-S129STOP::hphNT1	2A<br>S4A-B	this study
YSB1100	MATa HML hmrΔ::pRS bar1Δ::trp1 pGal-HO::ade3 hta2-S129STOP::kanMX4	2A<br>S4A-B	this study
YSB1096	MATa HML hmrΔ::pRS bar1Δ::trp1 pGal-HO::ade3 hta1-S129STOP::hphNT1 hta2-S129STOP::kanMX4	2A<br>S4A-B	this study
YSB408	MATa HML hmrΔ::pRS bar1Δ::trp1 pGal-HO::ade3 pSB143 dna2Δ::hphNT1 ddc1Δ::kanMX4	2D<br>S5A-B	this study
YSB171	MATa HML hmrΔ::pRS bar1Δ::trp1 pGal-HO::ade3 Ddc1-3FLAG::hphNT1	3C, I<br>S6A, F	this study
YSB243	MATa HML hmrΔ::pRS bar1Δ::trp1 pGal-HO::ade3 exo1::natNT2 sgs1::kanMX4 Ddc1-	3C<br>S6F	this study

	3FLAG::hphNT1		
YSB15	MATa HML hmrΔ::pRS bar1Δ::trp1 pGal-HO::ade3 Dpb11-3FLAG::natNT2	3A,C<br>S6A	this study
YSB381	MATa HML hmrΔ::pRS bar1Δ::trp1 pGal-HO::ade3 exo1::natNT2 sgs1::kanMX4 Dpb11-3FLAG::hphNT1	3C	this study
YSB9	MATa HML hmrΔ::pRS bar1Δ::trp1 pGal-HO::ade3 Rad9-3FLAG::hphNT1	3A,B,C<br>4C-D<br>S6A<br>S7A-B	this study
YSB146	MATa HML hmrΔ::pRS bar1Δ::trp1 pGal-HO::ade3 exo1::natNT2 sgs1::kanMX4 Rad9-3FLAG::hphNT1	3C	this study
YSB210	MATa HML hmrΔ::pRS bar1Δ::trp1 pGal-HO::ade3 Dpb11-3FLAG::natNT2 ddc1-T602A::hphNT1	3A	this study
YSB75	MatA ade3::pGAL::HO bar1Δ::TRP1 hmlΔ::pRS-1 hmrΔpRS-2 Rad9-3FLAG::hphNT1 ddc1-T602A:: natNT2	3A,B<br>S7B	this study
YSB218	MATa HML hmrΔ::pRS bar1Δ::trp1 pGal-HO::ade3 Rtt107-3FLAG::hphNT1	3A<br>S2E	this study
YSB219	MATa HML hmrΔ::pRS bar1Δ::trp1 pGal-HO::ade3 exo1Δ::natNT2 sgs1Δ::kanMX4 Rtt107-3FLAG::hphNT1	S2E	this study
YSB388	MatA ade3::pGAL::HO bar1Δ::TRP1 HML hmrΔpRS-2 Rtt107-3FLAG::natNT2 ddc1-T602A::natNT2	3A	this study
YSB1120	MatA ade3::pGAL::HO bar1Δ::TRP1 HML hmrΔpRS-2 Ddc1-3FLAG::hphNT1 exo1Δ::kanMX4	3I<br>S6F	this study
YSB1124	MatA ade3::pGAL::HO bar1Δ::TRP1 HML hmrΔpRS-2 Ddc1-3FLAG::hphNT1	3I<br>S6E	this study

	rad9Δ::natNT2		
YSB1122	MatA ade3::pGAL::HO bar1Δ::TRP1 HML hmrΔpRS-2 Ddc1-3FLAG::hphNT1 sgs1Δ::kanMX4	S6F	this study
YSB1105	MATa HML hmrΔ::pRS bar1Δ::trp1 pGal-HO::ade3 Ddc2-9myc::hphNT1	4A-B	this study
YSB1106	MATa HML hmrΔ::pRS bar1Δ::trp1 pGal-HO::ade3 Yiplac128-Ddc1-Rad9-3FLAG::LEU2 Ddc2-9myc::hphNT1	4A-B	this study
YSB1107	MATa HML hmrΔ::pRS bar1Δ::trp1 pGal-HO::ade3 Rad9-dpb11ΔN-3FLAG::kanMX4	4C-D S7A-B	this study
YSB1108	MATa HML hmrΔ::pRS bar1Δ::trp1 pGal-HO::ade3 Rad9-dpb11ΔN-3FLAG::kanMX4 ddc1-T602A::natNT2	S7A-B	this study
YCZ173	ade3::PGAL::HO ARS607::HOcs::KanMX bar1Δ::TRP1 hmlΔ::pRS-1 hmrΔ::pRS-2 matHOcsΔ::pBR-1	S2D	[2]
YSB517	MATa hmlΔ::prS hmrΔ::pRS bar1Δ::trp1 pGal-HO::ade3	S1C	[3]
YSB519	hmlΔ::prS hmrΔ::pRS bar1Δ::trp1 pGal-HO::ade3 exo1Δ::natNT2 sgs1Δ::kanMX4	S1C	this study
YSB522	Mata bar1Δ::trp1 pGal-HO::ade3	S1C	this study
YSB524	Mata bar1Δ::trp1 pGal-HO::ade3 exo1Δ::natNT2 sgs1Δ::kanMX4	S1C	this study
YSB1116	MATa HML hmrΔ::pRS bar1Δ::trp1 pGal-HO::ade3 pSB143 dna2Δ::hphNT1 ddc1Δ::kanMX4 tel1Δ::natNT2	S5A-B	this study
YSB397	MATa HML hmrΔ::pRS bar1Δ::trp1 pGal-HO::ade3 dpb11ΔC::hphNT1	S5D	this study
YSB412	MATa HML hmrΔ::pRS bar1Δ::trp1 pGal-HO::ade3 dpb11ΔC::hphNT1 tel1Δ::kanMX4	S5D	this study
YSB406	MATa HML hmrΔ::pRS bar1Δ::trp1 pGal-	S5D	this study

	HO::ade3 ddc1Δ::kanMX4		
YSB413	MATa HML hmrΔ::pRS bar1Δ::trp1 pGal-HO::ade3 ddc1Δ::kanMX4 tel1Δ::natNT2	S5C-D	this study
YSB407	MATa HML hmrΔ::pRS bar1Δ::trp1 pGal-HO::ade3 dna2Δ::hphNT1 Yiplac211-dna2-WYAA::URA3	S5D	this study
YSB414	MATa HML hmrΔ::pRS bar1Δ::trp1 pGal-HO::ade3 dna2Δ::hphNT1 Yiplac211-dna2-WYAA::URA3 tel1Δ::natNT2	S5C-D	this study
YSB1111	MATa HML hmrΔ::pRS bar1Δ::trp1 pGal-HO::ade3 pph3Δ::hphNT1	S5E-G	this study
YSB1112	MATa HML hmrΔ::pRS bar1Δ::trp1 pGal-HO::ade3 exo1Δ::natNT2 sgs1Δ::kanMX4 pph3Δ::hphNT1	S5E-G	this study
ML891-5A	MATa ADE2 ade3::pGAL::HO trp1-1 hmrΔpRS-2 DDC1-4ala-YFP RFA1-8ala-CFP RAD5	3D-H S6B-E	this study
ML896-8A	MAT**a** ADE2 ade3::pGAL::HO trp1-1 hmrΔpRS-2 DDC1-4ala-YFP RFA1-8ala-CFP RAD5 exo1Δ	3H S6B-E	this study
ML1096-17D	MAT**a** ADE2 ade3::pGAL::HO trp1-1 hmrΔpRS-2 DDC1-4ala-YFP RFA1-8ala-CFP RAD5 rad9Δ	3H S6B-E	this study
W5094-1C	MATa ADE2 trp1-1 LYS2 RAD52-YFP RAD5	3D-H S6B-E	[4]
ML187-1D	MATa ADE2 trp1-1 LYS2 RAD52-CFP RAD5	3D-H S6B-E	this study
ML1103-3B/3C	MATa ade2-1 trp1-1 LYS2 RAD5 Ddc1-4ala-YFP ura3::3xUra3-TetOx112 I-SceI (ura3-1) TetR-mRFP1 (iYGL119W) RFA1-8ala-CFP	S6H-I	this study
ML1103-6C/8D	MATa ade2-1 trp1-1 LYS2 RAD5 Ddc1-4ala-YFP ura3::3xUra3-TetOx112 I-SceI (ura3-1)	S6H-I	this study

	TetR-mRFP1 (iYGL119W) RFA1-8ala-CFP exo1::NatMX sgs1::HIS3		

Supplementary Table 2. Plasmids:

name	description
pSB251	Yiplac128-pDdc1-Ddc1-Rad9-3FLAG
pSB143	Yiplac211-pDna2o+t WY128,130AA

Supplementary Table 3. qPCR primer sequences:

name	directionality	sequence 5’-3’
0.6 kb in	5’-3’	CGAATTGGCTATACGGGACG
0.6 kb re	3’-5’	GGAGGCACCCAAGAAGGCG
1.1 kb in	5’-3’	CTCGGCATATTTGTATTAACCCACT
1.1 kb re	3’-5’	GTCCTCCGTCCAATCTGTGC
1.6 kb in	5’-3’	GACTCGTATGATGAAAGCC
1.6 kb re	3’-5’	GGTGAAGCAATTTTTACTGG
3 kb in	5’-3’	GATATTGGCCTAGAACTGCCGG
3 kb re	3’-5’	GCATGGGCACTTGCTAACCAAT
3.3 kb in	5’-3’	GCTTAGAAGGTGCGGTAGG
3.3 kb re	3’-5’	TGTTGTGGACAATGTTAACGA
4.7 kb in	5’-3’	CTTCATCTCATGCAAAGTGC
4.7 kb re	3’-5’	GGGGCAATTGGTAAATTGCG
6 kb in	5’-3’	CACTGCCTACTGTTGCCCC
6 kb re	3’-5’	GCCTATTGGGGTAATAGAC
7.6 kb in	5’-3’	CACCAAGAGGTAGTGTGAC
7.6 kb re	3’-5’	AGCCTTCTACGCCAAACCAG
8 kb in	5’-3’	GATGTTTACACAGGGCCCCC
8 kb re	3’-5’	CGTTCTTAGTGGTCTGGAGTTC
10 kb in	5’-3’	GAAGGAGACAGAGACAGAGGG
10 kb re	3’-5’	GAAGGGAGGCAAAGACAAGGAG
15 kb in	5’-3’	GTGGAGTTTGATGGTATCGACATC
15 kb re	3’-5’	CGTTGGAACCATCTTGAGCCTC
20 kb in	5’-3’	CATCGTTCTCTTCGTTCTCTTCG
20 kb re	3’-5’	CACAAATATCTCTTCTCGACGGC
24 kb in	5’-3’	CTCTGTGGGTATTTCCGTG
24 kb re	3’-5’	CTTGGCGCTACGATGTGC
28 kb in	5’-3’	CTGTGCTGTCTGCGCTGCATT
28 kb re	3’-5’	GACGAAGGAGACGAAAACCTCTTC
34 kb in	5’-3’	GGATGGATGGTTATGTTTCGGAAGG
34 kb re	3’-5’	CACCAGCAACTCTATCTTCGTTG
45 kb in	5’-3’	TCCAGTCGTCCAACTCTTGCC

45 kb re	3'-5'	CAAGATATTGAGCCTGGATGC
50 kb in	5'-3'	CATGTGGAGATTTCAGGAGAGG
50 kb re	3'-5'	GAAGAAAGTCGATCTGTTCC
55 kb in	5'-3'	AATAATGTCTGCCAGCAACGC
55 kb re	3'-5'	TGATGGATGTATGGACCAGAG
60 kb in	5'-3'	AGATCTATCTAATGAGCCGG
60 kb re	3'-5'	GATGGTGTTACCACCGTCGCTG
65 kb in	5'-3'	TCTTCCCGTGTTAACGACAAC
65 kb re	3'-5'	CAGAACTAGGATCAATCTTGG
70 kb in	5'-3'	AGCCCAGTAGTACTACCTCTC
70 kb re	3'-5'	ACAAACCTGTCAACACTGCG
75 kb in	5'-3'	CCCAAGCTCACAAATTAATATGGC
75 kb re	3'-5'	GCATCTGTAGTACCACTGCTCTTTG
ctrl ChrI in	5'-3'	TGGTCTGAGTTTCCAGTTCTTTGGT
ctrl ChrI re	3'-5'	AGCGTCCAAACTAAATGAGCAGTCT
ctrl ChrV in	5'-3'	TGATAGCTTCTGCAATCGTAGGGC
ctrl ChrV re	3'-5'	TGGATCACGGTGCTAAGGAGGTTA
ctrl ChrVI in	5'-3'	CTAAACGTGGCCGCATTTGGTAAG
ctrl ChrVI re	3'-5'	ATCATCGCCGATTGGATAAGGGTG
ctrl ChrXV in	5'-3'	ACTGCAACAAGACCTTCACTCAACT
ctrl ChrXV re	3'-5'	GCAGGATGGTTTTCTGGTGAGGA
HO intact in	5'-3'	GTGGCATTACTCCACTTCAAGTAAGAG
HO intact re	3'-5'	CTT CCC AAT ATC CGT CAC CAC G

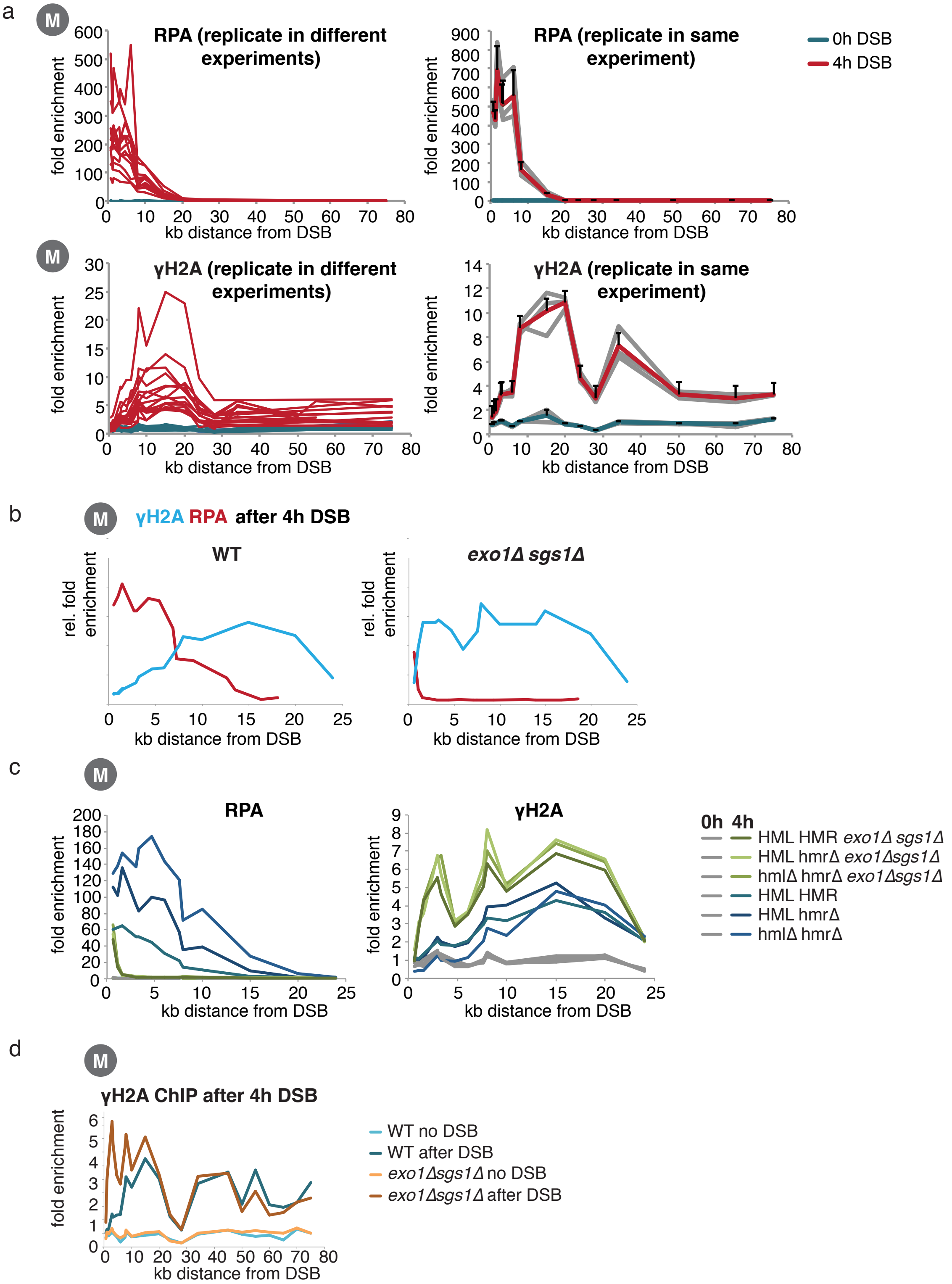
a
M
RPA (replicate in different experiments)
fold enrichment
kb distance from DSB
RPA (replicate in same experiment)
0h DSB
4h DSB
γH2A (replicate in different experiments)
γH2A (replicate in same experiment)
b
γH2A RPA after 4h DSB
WT
exo1Δ sgs1Δ
rel. fold enrichment
c
RPA
γH2A
0h 4h
HML HMR exo1Δ sgs1Δ
HML hmrΔ exo1Δsgs1Δ
hmlΔ hmrΔ exo1Δsgs1Δ
HML HMR
HML hmrΔ
hmlΔ hmrΔ
d
γH2A ChIP after 4h DSB
WT no DSB
WT after DSB
exo1Δsgs1Δ no DSB
exo1Δsgs1Δ after DSB

Supplementary Figure 1

γH2A phosphorylation is not correlated with ssDNA signal strength.

(A) ChIP signals for RPA (upper panels) and γH2A (lower panels) vary in fold enrichment of the ChIP signal between independent experiments (left panels), but are very reproducible in signal strength within one experiment (right panels). Right Panels: Mean (n =3) in red, error bars represent standard deviation.

(B) RPA and γH2A enrichments around a DSB are anti-correlated suggesting that resection as read by RPA enrichment evicts histone H2A and thereby the substrate for γH2A phosphorylation. Overlay of RPA (red) and γH2A (blue) ChIP signals after 4h of DSB in *WT* (left panel) and *exo1Δ sgs1Δ* cells (right panel) cells arrested in M phase.

(C) DNA end resection (left panel) and γH2A phosphorylation (right panel) were measured by ChIP in *WT* strains or *exo1Δ sgs1Δ* strains with both mating type loci deleted, intact *HML* or intact *HML* and *HMR*. All strains used in this study were *HML hmrΔ* if not indicated differently. Samples were analysed after 4 h of DSB induction.

(D) γH2A phosphorylation spreads over a large distance from a DSB (>75 kb) in *WT* and *exo1Δ sgs1Δ cells.* With the exception of the area of resection (Supplementary Fig.1A), highly similar γH2A phosphorylation is observed in presence and absence of resection. ChIP-qPCR analysis of *WT* cells or *exo1Δ sgs1Δ* cells after 4h of DSB induction.

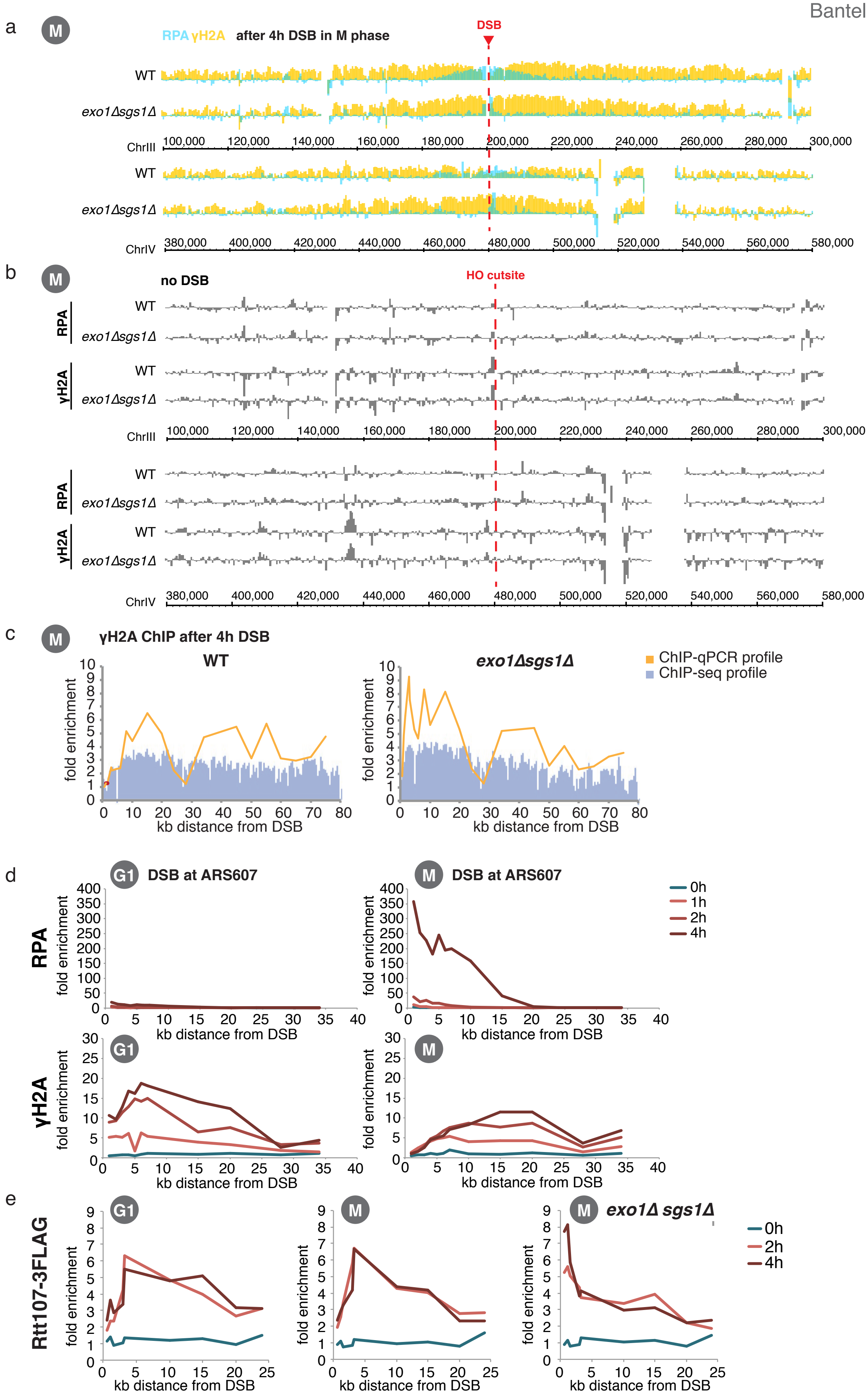
a
M
RPA γH2A after 4h DSB in M phase
DSB
WT
exo1Δsgs1Δ
ChrIII 100,000 120,000 140,000 160,000 180,000 200,000 220,000 240,000 260,000 280,000 300,000
WT
exo1Δsgs1Δ
ChrIV 380,000 400,000 420,000 440,000 460,000 480,000 500,000 520,000 540,000 560,000 580,000
b
M
no DSB
HO cutsite
RPA
γH2A
WT
exo1Δsgs1Δ
ChrIII 100,000 120,000 140,000 160,000 180,000 200,000 220,000 240,000 260,000 280,000 300,000
ChrIV 380,000 400,000 420,000 440,000 460,000 480,000 500,000 520,000 540,000 560,000 580,000
c
M
γH2A ChIP after 4h DSB
WT
exo1Δsgs1Δ
ChIP-qPCR profile
ChIP-seq profile
fold enrichment
kb distance from DSB
d
G1 DSB at ARS607
M DSB at ARS607
RPA
γH2A
fold enrichment
kb distance from DSB
0h
1h
2h
4h
e
Rtt107-3FLAG
G1
M
M exo1Δ sgs1Δ
fold enrichment
kb distance from DSB
0h
2h
4h

Supplementary Figure 2

γH2A phosphorylation is not correlated with ssDNA signal strength.

γH2A phosphorylation and RPA enrichment around two distinct DSBs anti-correlate in ChIP-seq experiments (A) and depend on the presence of the DSBs (B).

(A) Overlay of ChIP-seq profiles from the experiment shown in Fig. 1C. RPA enrichments are plotted in blue, γH2A phosphorylation is plotted in yellow. The positions of the HO-induced DSBs on chromosome 3 (upper two panels) and chromosome 4 (lower two panels) are indicated by the red dotted line. *WT* cells (upper traces) are compared with *exo1Δ sgs1Δ* mutant cells (lower traces).

(B) ChIP-seq profiles of the 0h time point before induction of DSB. Samples from the same experiment shown in Fig. 1C and Supplementary Fig. 2A, but shown is analysis of the enrichment of RPA (upper panel, respectively) and γH2A phosphorylation (lower panel, respectively).

(C) Overlay of ChIP-qPCR and ChIP-seq signals. *WT* and *exo1Δ sgs1Δ* cells were arrested in M phase and analysed for γH2A phosphorylation after 4h of DSB induction. qPCR signals (red) are from experiment in Fig. S1D, ChIP-seq data (blue) from experiment in Fig. 1C and Supplementary Fig. 2A and 2B.

(D) γH2A phosphorylation around a DSB is not showing a response that correlates with the ssDNA signal at a DSB induced next to ARS607. ChIP-qPCR analysis of strains carrying an ectopic HO cutsite next to ARS607 arrested in G1 (left panels) or M phase (right panels). RPA enrichment (upper panels) and γH2A phosphorylation (lower panels) were analysed at indicated times.

(E) Rtt107 associates with a DSB in a resection-independent manner. Rtt107 recruitment to a DSB was measured at indicated times in *WT* strains arrested in G1 (left panel) or in M phase (middle panel), or in *exo1Δ sgs1Δ* cells arrested in M phase (right panel). Rtt107 was tagged with a C-terminal 3FLAG tag and detected by a ChIP directed against the FLAG tag.

a

Model of the **local** checkpoint signalling circuit

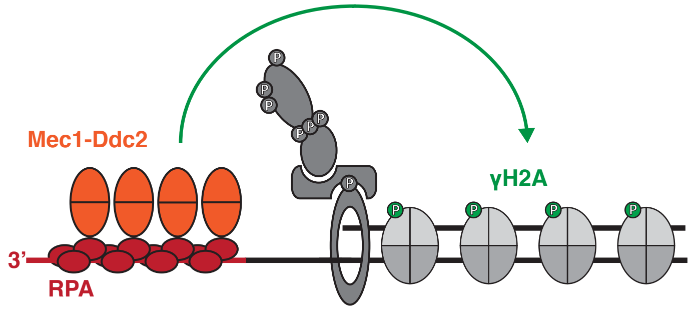

b

Model of the **global** checkpoint signalling circuit

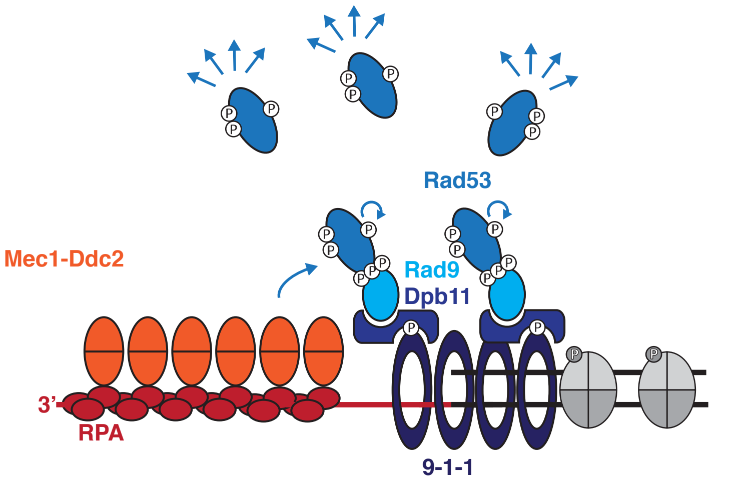

Supplementary Figure 3

DNA damage checkpoint signalling can be subdivided in two separate, Mec1-dependent signaling circuits. Models of local **(A)** and global **(B)** checkpoint signalling circuits.

(A) Mec1-Ddc2 (orange) bound to RPA (red) phosphorylates γH2A as a substrate of the local checkpoint signalling circuit (green) in a resection-independent manner.

(B) Mec1-Ddc2 (orange) bound to RPA (red) phosphorylates proteins of the 9-1-1 axis (Ddc1, Dpb11, Rad9 and Rad53, shades of blue) within the global checkpoint signalling circuit, which depends on DNA end resection and culminates in the activation of the Rad53 effector kinase.

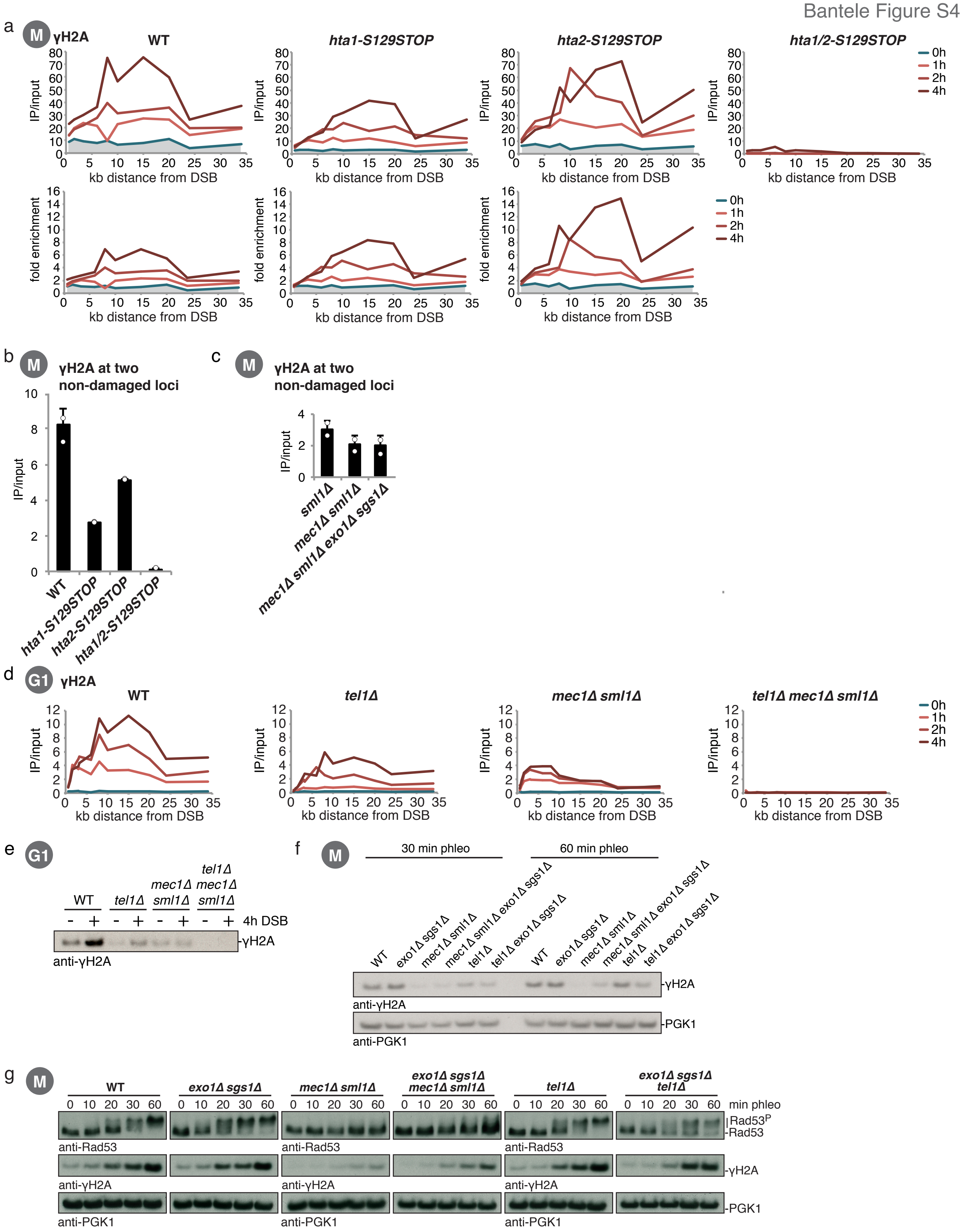
a
M γH2A
WT
hta1-S129STOP
hta2-S129STOP
hta1/2-S129STOP
0h
1h
2h
4h
IP/input
fold enrichment
kb distance from DSB
b
M γH2A at two non-damaged loci
IP/input
WT
hta1-S129STOP
hta2-S129STOP
hta1/2-S129STOP
c
M γH2A at two non-damaged loci
IP/input
sml1Δ
mec1Δ sml1Δ
mec1Δ sml1Δ exo1Δ sgs1Δ
d
G1 γH2A
WT
tel1Δ
mec1Δ sml1Δ
tel1Δ mec1Δ sml1Δ
IP/input
kb distance from DSB
e
G1
WT
tel1Δ
mec1Δ sml1Δ
tel1Δ mec1Δ sml1Δ
4h DSB
γH2A
anti-γH2A
f
M
30 min phleo
60 min phleo
WT
exo1Δ sgs1Δ
mec1Δ sml1Δ
mec1Δ sml1Δ exo1Δ sgs1Δ
tel1Δ
tel1Δ exo1Δ sgs1Δ
γH2A
anti-γH2A
PGK1
anti-PGK1
g
M
WT
exo1Δ sgs1Δ
mec1Δ sml1Δ
exo1Δ sgs1Δ mec1Δ sml1Δ
tel1Δ
exo1Δ sgs1Δ tel1Δ
0 10 20 30 60
min phleo
Rad53P
Rad53
anti-Rad53
γH2A
anti-γH2A
PGK1
anti-PGK1

Supplementary Figure 4

γH2A phosphorylation is dependent on Mec1 and Tel1 in G1 phase, but mainly determined by Mec1 in M phase.

(A) Specificity of the γH2A antibody in ChIP experiments. WT cells are compared to cells lacking the antibody epitope (*hta1-S129STOP hta2-S129STOP*), and mutants lacking the S129 phosphosite in one of the two H2A-coding genes, *HTA1,* and *HTA2*, respectively. Data shown are from experiment in Fig. 2A. Upper plots show the IP/input ratios of γH2A ChIPs, the lower plots show data normalized to non-damaged control loci. The grey area under the 0h curve visualizes strain-specific variations in the basal γH2A levels,.

(B,C) Basal γH2A levels at non-damaged loci in *hta1/hta2* mutants strains used in Fig. 2A and Supplementary Fig. 4A, and *mec1Δ sml1Δ* mutants from Fig. 2B. γH2A ChIP IP/input ratios from two loci on distinct, undamaged chromosomes are plotted. Error bars represent the standard deviation, dot plots represent replicates (n=2).

(D) Both Mec1 and Tel1 contribute to γH2A phosphorylation in G1. IP/input ratios of γH2A ChIP after indicated times of DSB induction in G1 phase. WT cells were compared to *tel1Δ*, *mec1Δ sml1Δ* and *mec1Δ sml1Δ tel1Δ* double mutant cells.

(E) γH2A phosphorylation after DSB induction in G1 phase is decreased in cells lacking Mec1 or Tel1, and abolished in *mec1Δ tel1Δ sml1Δ* cells. Western blot analysis of γH2A phosphorylation after 4h of HO induction in G1 phase cells.

(F-G) γH2A and Rad53 phosphorylation after phleomycin treatment in M phase is strongly decreased in cells lacking Mec1, but almost unaffected in cells lacking Tel1. **(F)** Western blot analysis of γH2A phosphorylation (upper panel) after 30 and 60 minutes of phleomycin treatment. Pgk1 served as loading control. Samples from same experiment as in (G). **(G)** Western blot analysis of Rad53 phosphorylation (upper panels, respectively), γH2A phosphorylation (middle panels, respectively) and Pgk1 as control (lower panels, respectively) in *WT* cells or *exo1Δ sgs1Δ*, *mec1Δ sml1Δ*, *exo1Δ sgs1Δ mec1Δ sml1Δ*, *tel1Δ*, *exo1Δ sgs1Δ tel1Δ* cells arrested in M phase and after treatment with 50 µg/ml phleomycin for the indicated times.

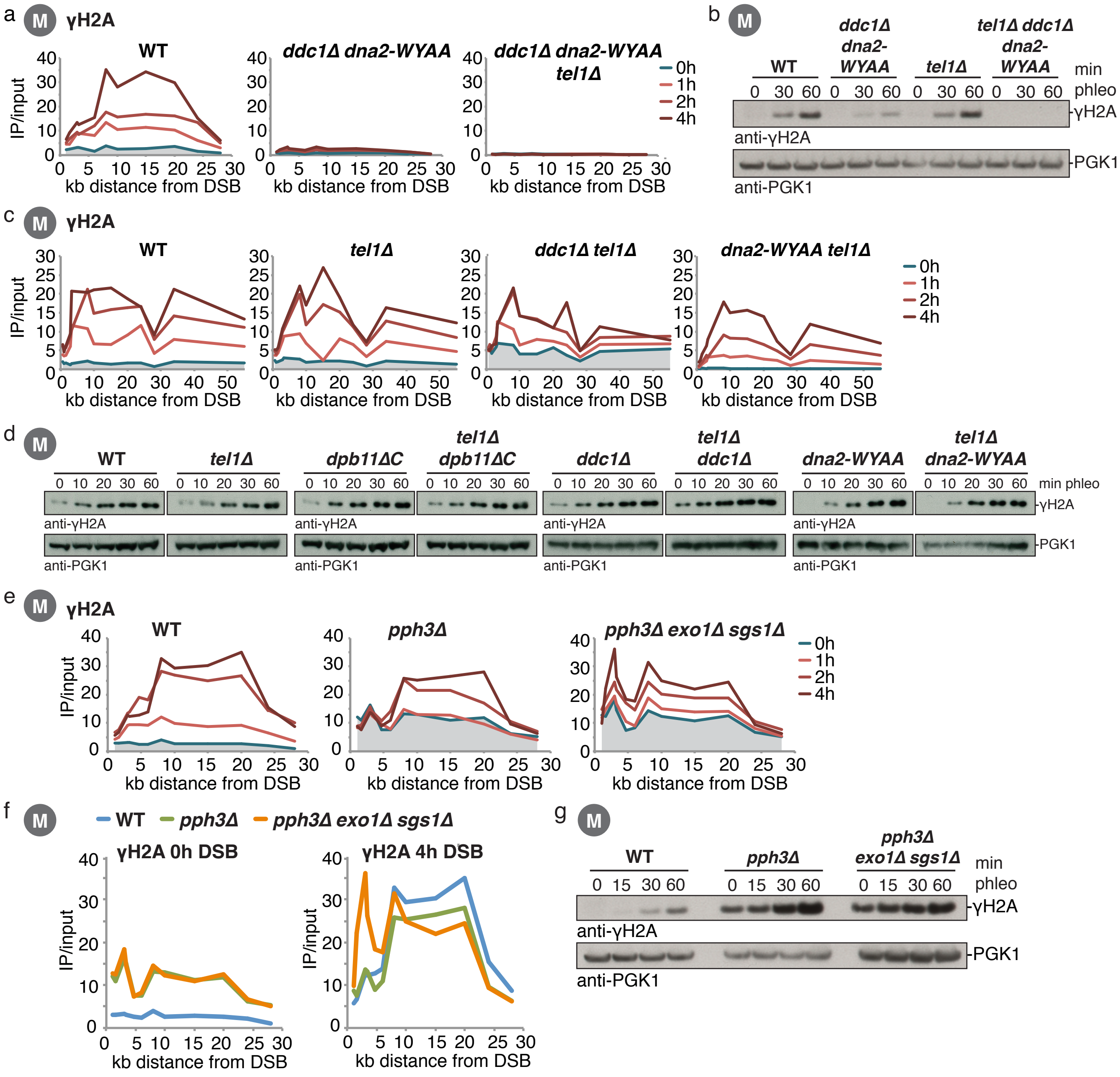
a
M
γH2A
WT
ddc1Δ dna2-WYAA
ddc1Δ dna2-WYAA tel1Δ
0h
1h
2h
4h
IP/input
kb distance from DSB
b
M
WT
ddc1Δ dna2-WYAA
tel1Δ
tel1Δ ddc1Δ dna2-WYAA
0 30 60
min phleo
γH2A
anti-γH2A
PGK1
anti-PGK1
c
M
γH2A
WT
tel1Δ
ddc1Δ tel1Δ
dna2-WYAA tel1Δ
0h
1h
2h
4h
IP/input
kb distance from DSB
d
M
WT
tel1Δ
dpb11ΔC
tel1Δ dpb11ΔC
ddc1Δ
tel1Δ ddc1Δ
dna2-WYAA
tel1Δ dna2-WYAA
0 10 20 30 60
min phleo
γH2A
anti-γH2A
PGK1
anti-PGK1
e
M
γH2A
WT
pph3Δ
pph3Δ exo1Δ sgs1Δ
0h
1h
2h
4h
IP/input
kb distance from DSB
f
M
WT
pph3Δ
pph3Δ exo1Δ sgs1Δ
γH2A 0h DSB
γH2A 4h DSB
IP/input
kb distance from DSB
g
M
WT
pph3Δ
pph3Δ exo1Δ sgs1Δ
0 15 30 60
min phleo
γH2A
anti-γH2A
PGK1
anti-PGK1

Supplementary Figure 5

γH2A phosphorylation requires Mec1 activation and is globally limited by the PP4 phosphatase.

(A,B) γH2A phosphorylation requires Mec1 activation. ChIP-qPCR analysis of γH2A phosphorylation in *WT* cells and *dna2-WYAA ddc1Δ* and *dna2-WYAA ddc1Δ tel1Δ* mutant cells arrested in M phase. IP/input ratios from γH2A ChIPs derived from the same experiment as in Fig. 2D **(A)** and γH2A phosphorylation in Western Blot after phleomycin treatment in M phase **(B)** are shown, Pgk1 served as loading control. Grey areas under the 0h curve in qPCR plots visualizes differences in basal γH2A levels before DSB induction.

(C,D) γH2A phosphorylation is not influenced by the absence of a specific Mec1 activator at an HO break **(C)** or after phleomycin treatment **(D)**. **(C)** ChIP-qPCR analysis of γH2A phosphorylation plotted as IP/input ratios in *WT* cells and *tel1Δ, ddc1Δ tel1Δ* and *dna2-WYAA tel1Δ* mutant cells. Grey areas under the 0h curve in qPCR plots visualize differences in basal γH2A levels before DSB induction. **(D)** Western blot analysis of γH2A phosphorylation (upper panels, respectively) and Pgk1 as a control (lower panels, respectively) in *WT* cells or *ddc1Δ, dpb11ΔC* or *dna2-WYAA* mutant cells, either in *WT* background or *tel1Δ* background arrested in M phase. Samples were analysed at indicated time points.

(E) Analysis of γH2A phosphorylation by ChIP-qPCR plotted as IP/input ratios at indicated time points in M phase. *WT* cells are compared to *pph3Δ* and *pph3Δ exo1Δ sgs1Δ* mutant cells. Grey areas under the 0h curve in qPCR plots visualize the differences in basal γH2A levels before DSB induction. **(F)** Overlay of γH2A signals after 0 hours (left plot) and 4 hours (right plot) of DSB induction, same experiment as in (E).

(G) γH2A phosphorylation is generally enhanced in absence of PP4. Western blot analysis of γH2A phosphorylation in WT and *pph3Δ* cells after phleomycin treatment, strains as in (E,F), Pgk1 serves as loading control.

Bantele Figure S6

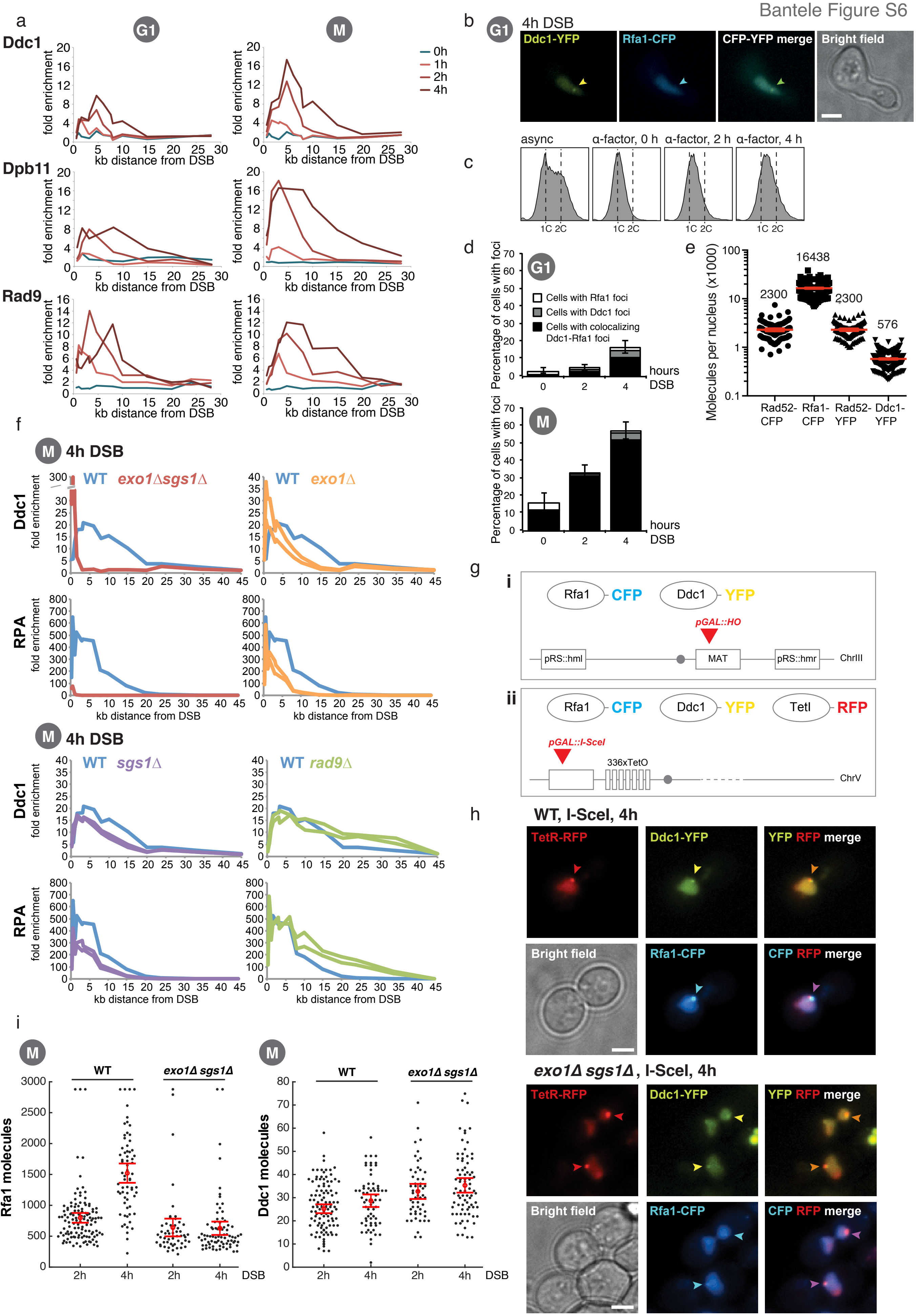

Supplementary Figure 6

Regulators of the global checkpoint circuit (9-1-1 axis) are cell cycle-regulated.

(A) Recruitment of 9-1-1 (Ddc1), Dpb11 and Rad9 is enhanced in M phase. ChIP-qPCR analysis of strains expressing FLAG-tagged checkpoint proteins in G1 (left) or M phase (right) at indicated times.

(B)-(E) Additional information related to the Ddc1- and RPA- foci analysis in Fig. 3D-H.

(B) Representative microscopy images from the Rfa1- and Ddc1-foci analysis in G1 before (upper row) and 4 hours after DSB induction (lower row). A DSB was induced at MAT in G1 using Gal-HO and cells were microscopically analysed for Ddc1-YFP foci (yellow, left panel) and Rfa1-CFP foci (blue, second to the left panel) at indicated times.

(C) FACS analysis before (asynchronous sample) and after addition of alpha-factor and 0, 2 and 4 hours of DSB induction.

(D) Ddc1 and RPA foci formation is more efficient in M phase than in G1. Same experiment as in Fig. 3D,E. Plots show the percentage of cells with foci at 0, 2 and 4 hours after DSB induction. After 4 hours, about 55% of M phase-arrested cells show foci, while only 15% of cells in G1 show foci. Error bars indicate mean with 95% confidence intervals (n=200-600).

(E) Normalization of Rfa1 and Ddc1 foci intensity against Rad52 as a standard. Error bars represent mean + 95% CI (n=124-371).

(F) RPA and Ddc1 recruitment to a DSB in resection mutants. ChIP-qPCR experiment against Ddc1 (upper panel) and RPA (lower panel) after 4 hours DSB in M phase, same experiment as in Fig. 3I, but additionally showing *sgs1Δ* (purple) and *exo1Δ sgs1Δ* (red) mutants.

(G) Cellular systems of DSB induction and foci visualization used in this study. **(i)** An HO break at the MAT locus on chrIII was used for experiments in Fig. 3D-H. **(ii)** a TetO-TetI-RFP-labeled I-SceI break on chrV was used in Supplementary Fig. 6H-I.

(H) Representative microscopy images from the Rfa1- and Ddc1-foci colocalization with an RFP-labeled I-SceI DSB (Supplementary Fig. 6Gii). M phase-arrested *WT* cells (upper panels) or *exo1Δ sgs1Δ* cells (lower panels) were analysed for Ddc1-YFP foci (yellow, upper panels, middle and right), Rfa1-CFP foci (blue, lower panels, middle and right), and TetR-RFP foci (red, left panels). Scale bars represent 3 μm.

(I) RPA and Ddc1 recruitment to a DSB is regulated differently in the absence of long-range resection. Scatter plot depicting RPA (left graph) or Ddc1 (right graph) molecules per focus. Analysis as in Fig. 3F and H but in the I-SceI DSB system (G). *WT* and *exo1Δ sgs1Δ* mutant cells were compared. Error bars represent the mean + 95% CI (n=54-124).

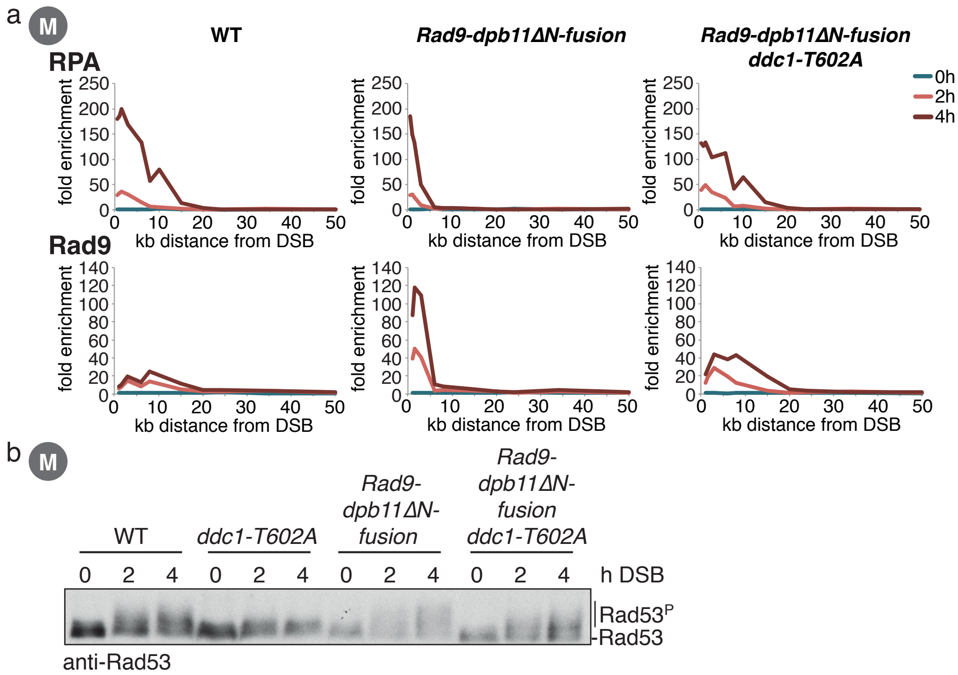
a
M
WT
Rad9-dpb11ΔN-fusion
Rad9-dpb11ΔN-fusion ddc1-T602A
RPA
fold enrichment
kb distance from DSB
0h
2h
4h
Rad9
b
M
WT
ddc1-T602A
Rad9-dpb11ΔN-fusion
Rad9-dpb11ΔN-fusion ddc1-T602A
0 2 4 0 2 4 0 2 4 0 2 4 h DSB
Rad53P
Rad53
anti-Rad53

Supplementary Figure 7

The action of the Rad9-dpb11ΔN fusion is influenced by Ddc1 phosphorylation.

(A) ChIP-qPCR analysis of DNA resection by RPA ChIP (upper panels) and Rad9 recruitment (lower panels) in *WT* cells, *Rad9-dpb11ΔN fusion* cells and *ddc1-T602A Rad9-dpb11ΔN fusion* cells. All strains were arrested in M phase and samples were taken at indicated time points. For detection, Rad9 and the Rad9 fusion were tagged C-terminally with a 3FLAG tag and the ChIPs were directed against the 3FLAG tag. The data in the left and middle panels is identical to the data shown in Fig. 4C.

(B) Increased Rad53 activation by the Rad9-dpb11ΔN fusion is dependent on the Ddc1 phosphorylation The same strains as in (A) were analysed for checkpoint activation after DSB induction in a Rad53 Western Blot, including a *ddc1-T602A* mutant strain. The Western Blot samples are from the same experiment as shown in Fig. 4D.

Supplementary References

1. Thomas, B. J. & Rothstein, R. Elevated Recombination Rates in Transcriptionally Active DNA. *Cell* 56, 619–630 (1989).

2. Zierhut, C. & Diffley, J. F. X. Break dosage, cell cycle stage and DNA replication influence DNA double strand break response. *The EMBO Journal* 27, 1875–1885 (2008).

3. Bantele, S., Ferreira, P., Gritenaite, D., Boos, D. & Pfander, B. Targeting of the Fun30 nucleosome remodeller by the Dpb11 scaffold facilitates cell cycle-regulated DNA end resection. *Elife* 6, e21687 (2017).

4. Lisby, M., Rothstein, R. & Mortensen, U. H. Rad52 forms DNA repair and recombination centers during S phase. *Proc. Natl. Acad. Sci. U.S.A.* 98, 8276–8282 (2001).
