# Supplementary material for: Quantitative sensing and signalling of single-stranded DNA during the DNA damage response
Source: Nat Commun. 2019 Feb 26;10:944. doi: 10.1038/s41467-019-08889-5 (PMC6391461; doi:10.1038/s41467-019-08889-5)
Supplement: Supplementary file 3 — Reporting Summary [file 41467_2019_8889_MOESM3_ESM.pdf]

nature research

Corresponding author(s): Pfander, Boris

Last updated by author(s): NCOMMS-18-10108-T

Reporting Summary

Nature Research wishes to improve the reproducibility of the work that we publish. This form provides structure for consistency and transparency in reporting. For further information on Nature Research policies, see Authors & Referees and the Editorial Policy Checklist.

Statistics

For all statistical analyses, confirm that the following items are present in the figure legend, table legend, main text, or Methods section.

n/a	Confirmed	
☐	☒	The exact sample size (*n*) for each experimental group/condition, given as a discrete number and unit of measurement
☒	☐	A statement on whether measurements were taken from distinct samples or whether the same sample was measured repeatedly
☒	☐	The statistical test(s) used AND whether they are one- or two-sided<br>*Only common tests should be described solely by name; describe more complex techniques in the Methods section.*
☒	☐	A description of all covariates tested
☒	☐	A description of any assumptions or corrections, such as tests of normality and adjustment for multiple comparisons
☐	☒	A full description of the statistical parameters including central tendency (e.g. means) or other basic estimates (e.g. regression coefficient) AND variation (e.g. standard deviation) or associated estimates of uncertainty (e.g. confidence intervals)
☒	☐	For null hypothesis testing, the test statistic (e.g. *F*, *t*, *r*) with confidence intervals, effect sizes, degrees of freedom and *P* value noted<br>*Give P values as exact values whenever suitable.*
☒	☐	For Bayesian analysis, information on the choice of priors and Markov chain Monte Carlo settings
☒	☐	For hierarchical and complex designs, identification of the appropriate level for tests and full reporting of outcomes
☒	☐	Estimates of effect sizes (e.g. Cohen's *d*, Pearson's *r*), indicating how they were calculated

Our web collection on statistics for biologists contains articles on many of the points above.

Software and code

Policy information about availability of computer code

Data collection	NA
Data analysis	NA

For manuscripts utilizing custom algorithms or software that are central to the research but not yet described in published literature, software must be made available to editors/reviewers. We strongly encourage code deposition in a community repository (e.g. GitHub). See the Nature Research guidelines for submitting code & software for further information.

Data

Policy information about availability of data

All manuscripts must include a data availability statement. This statement should provide the following information, where applicable:

- Accession codes, unique identifiers, or web links for publicly available datasets
- A list of figures that have associated raw data
- A description of any restrictions on data availability

All yeast strains and plasmids will be made available on request. ChIP-seq data is available from NCBI GEO (GSE124948). A source data file is included in the submission, containing unprocessed and uncropped Western blot scans from all main figures.

Field-specific reporting

Please select the one below that is the best fit for your research. If you are not sure, read the appropriate sections before making your selection.

☒ Life sciences ☐ Behavioural & social sciences ☐ Ecological, evolutionary & environmental sciences

Life sciences study design

All studies must disclose on these points even when the disclosure is negative.

Sample size	except imaging experiments, all data is from ensemble experiments from >2x10e7 cells; for imaging analysis at least 100 cells were analysed
Data exclusions	NA
Replication	All experiments were repeated with different numbers of repetitions (see reproducibility statement in the methods section) to confirm the reproducibility of the data.
Randomization	NA
Blinding	NA

Reporting for specific materials, systems and methods

We require information from authors about some types of materials, experimental systems and methods used in many studies. Here, indicate whether each material, system or method listed is relevant to your study. If you are not sure if a list item applies to your research, read the appropriate section before selecting a response.

Materials & experimental systems

n/a	Involved in the study	
☐	☒	Antibodies
☒	☐	Eukaryotic cell lines
☒	☐	Palaeontology
☒	☐	Animals and other organisms
☒	☐	Human research participants
☒	☐	Clinical data

Methods

n/a	Involved in the study	
☐	☒	ChIP-seq
☒	☐	Flow cytometry
☒	☐	MRI-based neuroimaging

Antibodies

Antibodies used

RPA - AS07-214, Agrisera - https://www.agrisera.com/en/artiklar/rfa-bakers-yeast-replication-factor-a-.html
FLAG - M2 - Sigma - https://www.sigmaaldrich.com/catalog/product/sigma/f1804?lang=de&region=DE
Rad53 - ab104232, abcam - https://www.abcam.com/rad53-antibody-ab104232.html
gammaH2A - ab15083, abcam - https://www.abcam.com/histone-h2a-phospho-s129-antibody-ab15083.html
Cdc48 - Jentsch Lab - Richly et al., Cell, 2005
Pgk1 - 22D5C8, Invitrogen - https://www.thermofisher.com/antibody/product/PGK1-Antibody-Polyclonal/PA5-28612

Validation

RPA - AS07-214, Agrisera:
Deshpande et al. (2017). Structural Basis of Mec1-Ddc2-RPA Assembly and Activation on Single-Stranded DNA at Sites of Damage. Mol Cell. 2017 Oct 19;68(2):431-445.e5. doi: 10.1016/j.molcel.2017.09.019.
Chen et al. (2017). Dihydrocoumarin, an HDAC Inhibitor, Increases DNA Damage Sensitivity by Inhibiting Rad52. Int J Mol Sci. 2017 Dec 7;18(12). pii: E2655. doi: 10.3390/ijms18122655.
Yeeles et al. (2015). Regulated eukaryotic DNA replication origin firing with purified proteins. Nature. 2015 Mar 4. doi: 10.1038/nature14285.
Holstein et al. (2014). Interplay between Nonsense-Mediated mRNA Decay and DNA Damage Response Pathways Reveals that Stn1 and Ten1 Are the Key CST Telomere-Cap Components. Cell Rep. 2014 May 22;7(4):1259-69. doi: 10.1016/j.celrep.2014.04.017. Epub 2014 May 15. (ChIP)
Deng et al. (2014). RPA antagonizes microhomology-mediated repair of DNA double-strand breaks. Nat Struct Mol Biol. 2014 Mar 9. doi: 10.1038/nsmb.2786. (western blot)
Bentensen et al. (2013). MRX protects fork integrity at protein–DNA barriers, and its absence causes checkpoint activation dependent on chromatin contex. Nucleic Acids Res. 2013 Mar 1;41(5):3173-89. doi: 10.1093/nar/gkt051. (ChIP)
FLAG - M2 - Sigma:
ADAR2 regulates RNA stability by modifying access of decay-promoting RNA-binding proteins Aparna A, et.al Nucleic Acids Research 45(7), 4189-4201, (2017)
MCPIP1, alias Regnase-1 binds and cleaves mRNA of C/EBP? Barbara Lipert, et.al PLoS ONE , -, (2017)
Histone H1 Limits DNA Methylation in Neurospora crassa Michael Seymour, et.al G3 (Bethesda, Md.) , -, (2016)
HSV-2 glycoprotein gD targets the CC domain of tetherin and promotes tetherin degradation via lysosomal pathway Yalan Liu,et.al Virology , 154-154, (2016)
Rad53 - ab104232, abcam:
Rodrigues J et al. Vps74 Connects the Golgi Apparatus and Telomeres in Saccharomyces cerevisiae. G3 (Bethesda) 8:1807-1816 (2018). PubMed: 29593073Gobbini E et al. The MRX complex regulates Exo1 resection activity by altering DNA end structure. EMBO J 37:N/A (2018). PubMed: 29925516Colombo CV et al. The RNA binding protein Npl3 promotes resection of DNA double-

strand breaks by regulating the levels of Exo1. Nucleic Acids Res 45:6530-6545 (2017).
gammaH2A - ab15083, abcam:
Peng XP et al. Acute Smc5/6 depletion reveals its primary role in rDNA replication by restraining recombination at fork pausing sites. PLoS Genet 14:e1007129 (2018). PubMed: 29360860Sein H et al. Rpb9-deficient cells are defective in DNA damage response and require histone H3 acetylation for survival. Sci Rep 8:2949 (2018). PubMed: 29440683Pal S et al. Impaired cohesion and homologous recombination during replicative aging in budding yeast. Sci Adv 4:eaaq0236 (2018). PubMed: 29441364Becker JR et al. Flap endonuclease overexpression drives genome instability and DNA damage hypersensitivity in a PCNA-dependent manner. Nucleic Acids Res 46:5634-5650 (2018). PubMed: 29741650Kim JC et al. The role of break-induced replication in large-scale expansions of (CAG)n/(CTG)n repeats. Nat Struct Mol Biol 24:55-60 (2017).
Cdc48 - Jentsch Lab - Richly et al., Cell, 2005
Pgk1 - 22D5C8, Invitrogen:
Yeast. 2017 Sep;34(9):371-382. doi: 10.1002/yea.3239. Epub 2017 Jun 30.
Cost-effective and rapid lysis of Saccharomyces cerevisiae cells for quantitative western blot analysis of proteins, including phosphorylated eIF2α.
Lee SJ1, Ramesh R1, de Boor V1, Gebler JM1, Silva RC1, Sattlegger E1.
Mol Cell. 2017 Mar 2;65(5):787-800.e5. doi: 10.1016/j.molcel.2017.01.005. Epub 2017 Feb 9.
Nuclear RNA Decay Pathways Aid Rapid Remodeling of Gene Expression in Yeast.
Bresson S1, Tuck A2, Staneva D1, Tollervey D3.
J Biol Chem. 2017 Feb 3;292(5):1815-1825. doi: 10.1074/jbc.M116.766980. Epub 2016 Dec 20.
The Assembly Factor Pet117 Couples Heme a Synthase Activity to Cytochrome Oxidase Assembly.
Taylor NG1, Swenson S2, Harris NJ1, Germany EM2, Fox JL3, Khalimonchuk O4.

ChIP-seq

Data deposition

☒ Confirm that both raw and final processed data have been deposited in a public database such as GEO.

☐ Confirm that you have deposited or provided access to graph files (e.g. BED files) for the called peaks.

Data access links
May remain private before publication.

GSE124948 [https://www.ncbi.nlm.nih.gov/geo/query/acc.cgi?acc=GSE124948]
IP sample WT 0h RPA
GSM3559929 [https://www.ncbi.nlm.nih.gov/geo/query/acc.cgi?acc=GSM3559929]
IP sample WT 4h RPA
GSM3559930 [https://www.ncbi.nlm.nih.gov/geo/query/acc.cgi?acc=GSM3559930]
IP sample exo1Δ sgs1Δ 0h RPA
GSM3559931 [https://www.ncbi.nlm.nih.gov/geo/query/acc.cgi?acc=GSM3559931]
IP sample exo1Δ sgs1Δ 4h RPA
GSM3559932 [https://www.ncbi.nlm.nih.gov/geo/query/acc.cgi?acc=GSM3559932]
IP sample WT 0h γH2A
GSM3559933 [https://www.ncbi.nlm.nih.gov/geo/query/acc.cgi?acc=GSM3559933]
IP sample WT 4h γH2A
GSM3559934 [https://www.ncbi.nlm.nih.gov/geo/query/acc.cgi?acc=GSM3559934]
IP sample exo1Δ sgs1Δ 0h γH2A
GSM3559935 [https://www.ncbi.nlm.nih.gov/geo/query/acc.cgi?acc=GSM3559935]
IP sample exo1Δ sgs1Δ 4h γH2A
GSM3559936 [https://www.ncbi.nlm.nih.gov/geo/query/acc.cgi?acc=GSM3559936]
Input DNA WT 0h
GSM3559937 [https://www.ncbi.nlm.nih.gov/geo/query/acc.cgi?acc=GSM3559937]
Input DNA WT 4h
GSM3559938 [https://www.ncbi.nlm.nih.gov/geo/query/acc.cgi?acc=GSM3559938]
Input DNA exo1Δ sgs1Δ 0h
GSM3559939 [https://www.ncbi.nlm.nih.gov/geo/query/acc.cgi?acc=GSM3559939]
Input DNA exo1Δ sgs1Δ 4h
GSM3559940 [https://www.ncbi.nlm.nih.gov/geo/query/acc.cgi?acc=GSM3559940]

Files in database submission

NA

Genome browser session
(e.g. UCSC)

NA

Methodology

Replicates

The ChIP-seq experiment shown in Fig. 1C and Supplementary Fig. 2A-B was performed more than 3 times using a qPCR read-out and samples were sent for sequencing once.

Sequencing depth

50 bp read-length, single-end reads

Antibodies

RPA - AS07-214, Agrisera - https://www.agrisera.com/en/artiklar/rfa-bakers-yeast-replication-factor-a-.html
gammaH2A - ab15083, abcam - https://www.abcam.com/histone-h2a-phospho-s129-antibody-ab15083.html

Peak calling parameters

NA

Data quality	Raw datas quality was check using the fastqc tool (v. 0.11.2)
Software	Raw data was mapped to the S. cerevisiae genome, excluding multimapped reads (option -m 1).<br>Using the R (v. 3.22) package GenomicRanges (v. 1.22) the files converted into binned table with a windows size of 1000bp.<br>the regions in the bam files overlapped the binned regions of the genome were normalized using the TMM normalisation from the edgeR packge (v. 3.12.0)<br>the table were than converted into wig files for better visulization<br>Ensembl, build R64-1-1<br>wig files were generated using the R software suite.
