# Supplementary material for: Quantitative sensing and signalling of single-stranded DNA during the DNA damage response
Source: Nat Commun. 2019 Feb 26;10:944. doi: 10.1038/s41467-019-08889-5 (PMC6391461; doi:10.1038/s41467-019-08889-5)
Supplement: Supplementary file 4 — Source Data [file 41467_2019_8889_MOESM4_ESM.pdf]

Source data file to

Quantitative sensing and signalling of single-stranded DNA during the DNA damage response

Bantele et al.

Contents:

Uncropped and unprocessed scans of Western Blots presented in Fig. 1B,E; Fig. 3B and Fig. 4B,D.

Source data to Fig. 1B

Rad53 ▶

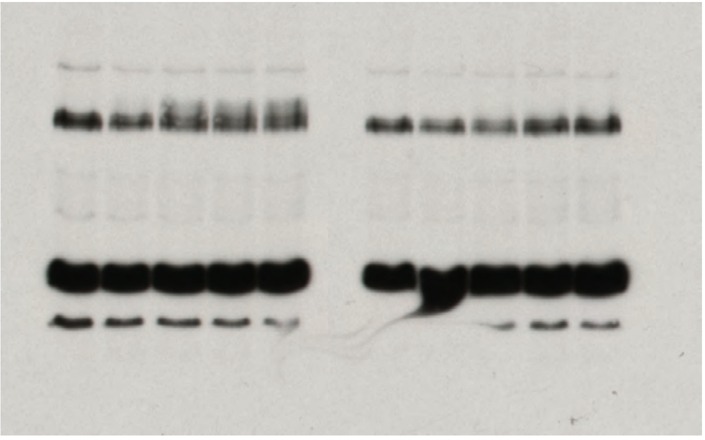

rabbit anti-Rad53
abcam ab104232
1:4000

Cdc48 ▶

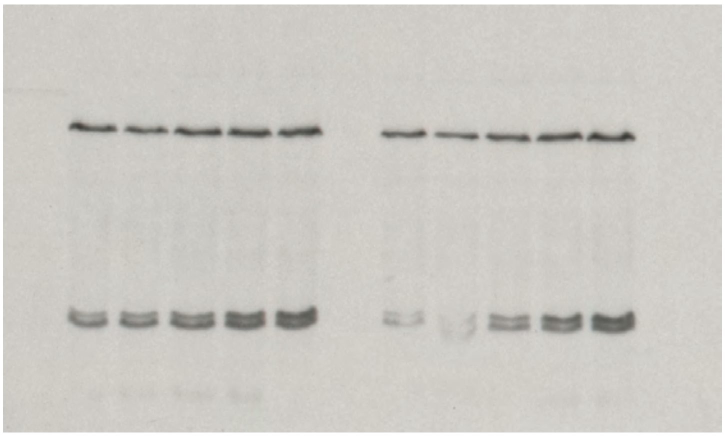

rabbit anti-Cdc48
Jentsch lab
1:10 000

Source data to Fig. 1E

Rad53 ▶

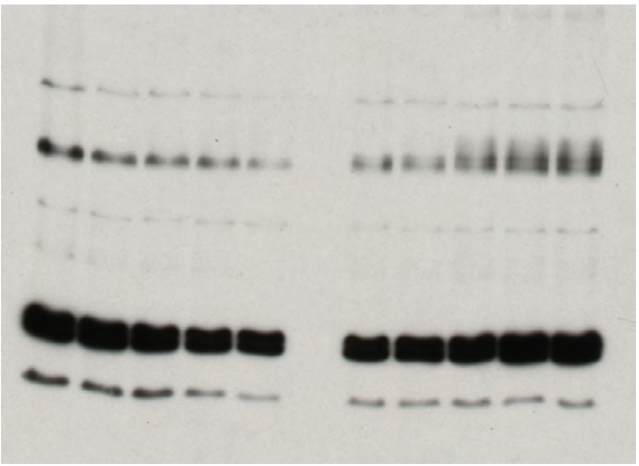

rabbit anti-Rad53
abcam ab104232
1:4000

Cdc48 ▶

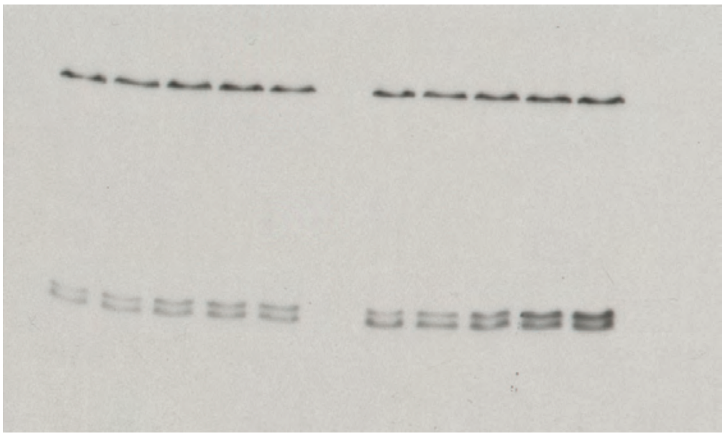

rabbit anti-Cdc48
Jentsch lab
1:10 000

Rad53 ▶

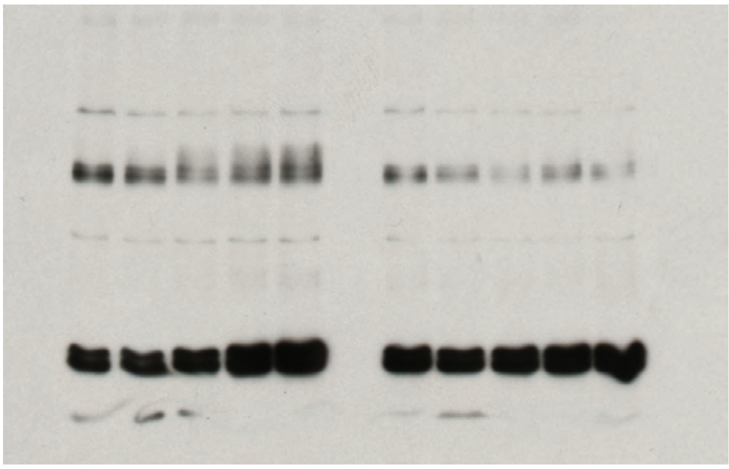

rabbit anti-Rad53
abcam ab104232
1:4000

Cdc48 ▶

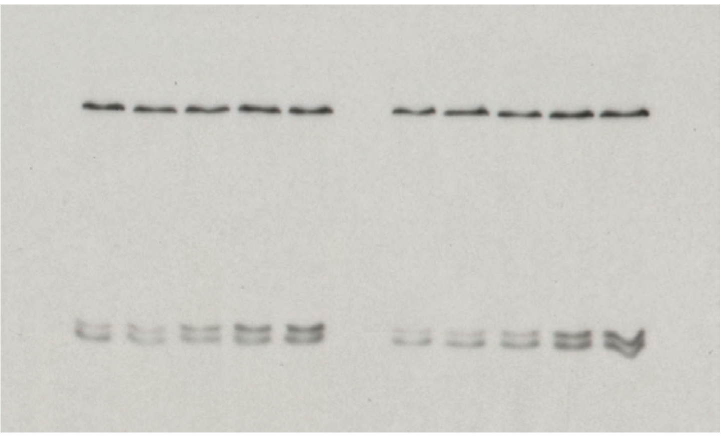

rabbit anti-Cdc48
Jentsch lab
1:10 000

Source data to Fig. 4B

Rad53 ▶

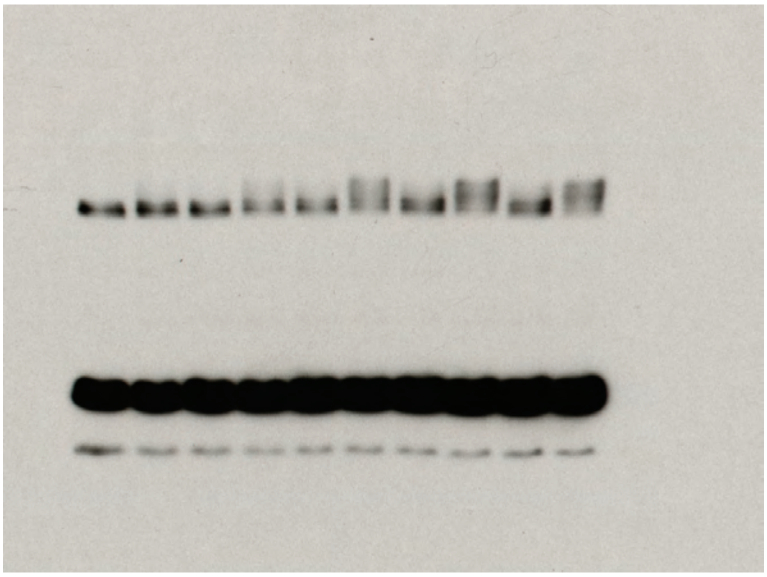

rabbit anti-Rad53
abcam ab104232
1:4000

Cdc48 ▶

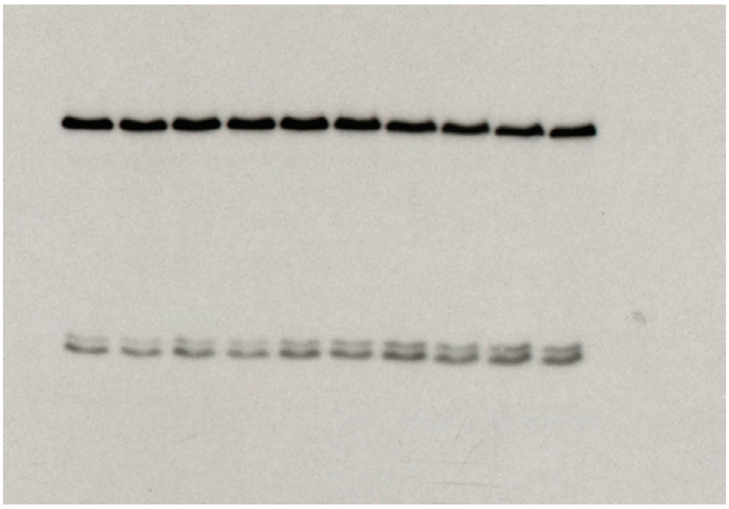

rabbit anti-Cdc48
Jentsch lab
1:10 000

Source data to Fig. 4D

Rad53 ▶

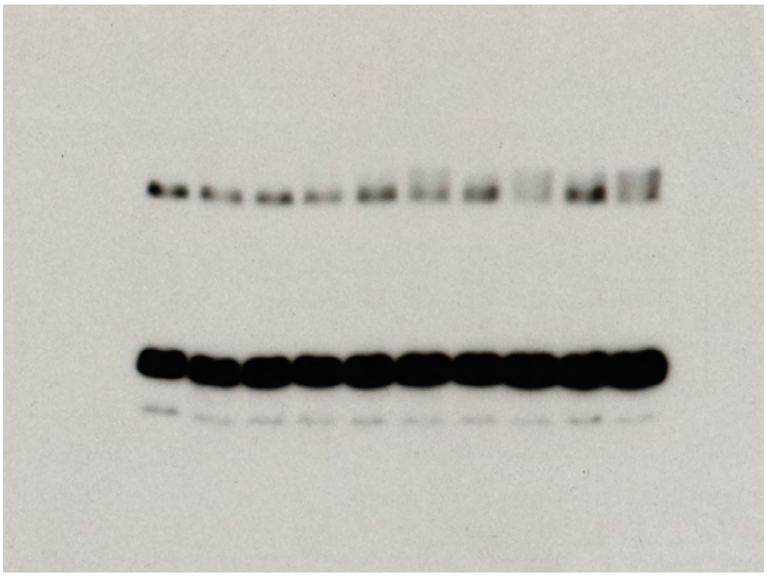

rabbit anti-Rad53
abcam ab104232
1:4000

Cdc48 ▶

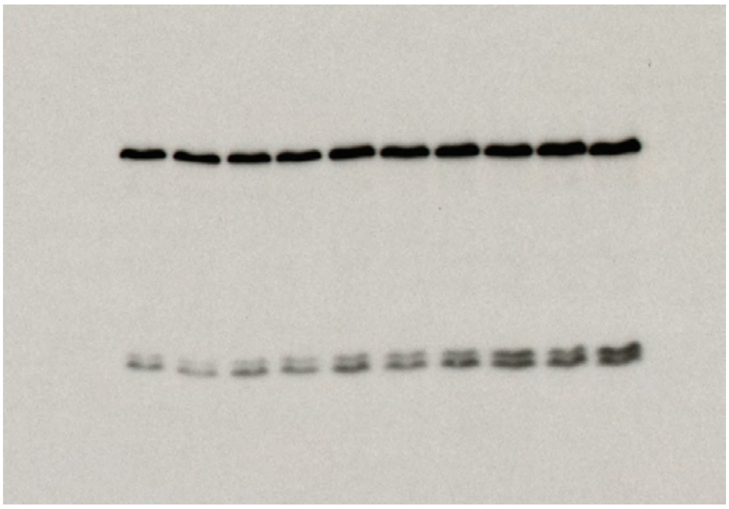

rabbit anti-Cdc48
Jentsch lab
1:10 000
